# Does a plant‐eating insect's diet govern the evolution of insecticide resistance? Comparative tests of the pre‐adaptation hypothesis

**DOI:** 10.1111/eva.12579

**Published:** 2017-12-18

**Authors:** Nate B. Hardy, Daniel A. Peterson, Laura Ross, Jay A. Rosenheim

**Affiliations:** ^1^ Department of Entomology and Plant Pathology Auburn University Auburn AL USA; ^2^ Graduate Program in Organismic & Evolutionary Biology Department of Biology University of Massachusetts Amherst MA USA; ^3^ School of Biological Sciences Institute of Evolutionary Biology University of Edinburgh Edinburgh UK; ^4^ Department of Entomology and Nematology University of California Davis CA USA

**Keywords:** generalized linear mixed models, pesticide resistance, phylogeny, plant–insect interactions

## Abstract

According to the pre‐adaptation hypothesis, the evolution of insecticide resistance in plant‐eating insects co‐opts adaptations that initially evolved during chemical warfare with their host plants. Here, we used comparative statistics to test two predictions of this hypothesis: (i) Insects with more diverse diets should evolve resistance to more diverse insecticides. (ii) Feeding on host plants with strong or diverse qualitative chemical defenses should prime an insect lineage to evolve insecticide resistance. Both predictions are supported by our tests. What makes this especially noteworthy is that differences in the diets of plant‐eating insect species are typically ignored by the population genetic models we use to make predictions about insecticide resistance evolution. Those models surely capture some of the differences between host‐use generalists and specialists, for example, differences in population size and migration rates into treated fields, but they miss other potentially important differences, for example, differences in metabolic diversity and gene expression plasticity. Ignoring these differences could be costly.

## 1 INTRODUCTION

Many insect species eat and spoil our crops. For eighty years, we have been killing them with synthesized organic chemicals. And for just as long—although it took us a few years to notice—insect pest populations have been evolving resistance to these chemicals (Denholm, Devine, & Williamson, 2002). The evolution of insecticide resistance makes farming less productive, less sustainable, more expensive, and more harmful to the environment. What determines how rapidly insecticide resistance evolves? What limits the breadth of insecticide resistance evolution? What factors govern the evolution of insecticide resistance in agricultural pests? Here we consider one possibility: the evolutionary history of host‐plant use.

In the pre‐adaptation hypothesis (Gordon, 1961), plant‐eating insects survive exposure to synthetic insecticides using physiological systems that initially evolved to survive exposure to naturally produced insecticides, that is, the defensive chemicals of their host plants. This hypothesis is consistent with numerous observations of cross‐resistance: In many cases, a by‐product of adaptation to a toxic host plant is reduced insecticide sensitivity (Bass et al., 2013; Dermauw et al., 2013; Gould, Carroll, & Futuyma, 1982). (As an aside, it would be interesting to see whether the reverse is true. Does selecting for insecticide resistance improve performance on toxic hosts?) The pre‐adaptation hypothesis is also consistent with our understanding of metabolic resistance; detoxification of insecticides and plant defensive chemicals rely on many of the same metabolic pathways (Bass et al., 2013, 2014; Daborn et al., 2002; Li, Schuler, & Berenbaum, 2007) and induce similar changes in gene expression (Dermauw et al. 2013).

But does the pre‐adaptation hypothesis actually do a good job of predicting the evolution of insecticide resistance? After all, it is a simple idea that ignores the population genetic factors that we usually think about when we think about evolutionary dynamics (e.g., Caprio & Tabashnik, 1992; Carrière et al., 2012; Georghiou & Taylor, 1977; Peck, Gould, & Ellner, 1999; Sisterson, Antilla, Carrière, Ellers‐Kirk, & Tabashnik, 2004; Stratonovitch, Elias, Denholm, Slater, & Semenov, 2014). As it stands, there has been one published comparative statistical test. Rosenheim, Johnson, Mau, Welter, and Tabashnik (1996) used the pre‐adaptation hypothesis to make this prediction: Plant‐eating insect lineages with a history of exposure to higher doses of plant defensive chemicals should more readily evolve resistance to insecticides. Some plant tissues are more defended than others; for example, phloem and xylem sap are thought to have much lower concentrations of defensive chemicals than leaf parenchyma cells (Douglas, 2006). On this basis, Rosenheim et al. predicted that sapsuckers should evolve less resistance than leaf‐chewers. Their comparative analysis supported this prediction. However, it had an important shortcoming. Specifically, it did not account for phylogenetic pseudo‐replication.

If we observe that two species share a trait, it could be that they each evolved that trait independently, through parallel responses to selection. Alternatively, it could be that they inherited the trait from a common ancestor. Ignoring the possibility of this inheritance causes us to inflate our counts of independent observations of a trait, which biases any statistical analysis of how that trait is associated with other traits. Rosenheim et al. (1996) were aware of the problem of phylogenetic pseudo‐replication, and because of it, they strongly hedged their conclusions: Something was decreasing the odds of sapsuckers evolving insecticide resistance, but they could not say whether it was because of their trophic mode, or any other pre‐adaptation.

Sapsucking appears to have evolved only once in plant‐eating insects (Douglas, 2006). Without replication, we cannot use comparative analyses to gauge its effects on insecticide resistance. But the pre‐adaptation hypothesis makes other predictions that have yet to be tested and that can be with statistical approaches that account for phylogenetic pseudo‐replication. For one, as noted by Rosenheim et al. (1996), it predicts the evolution of broad insecticide resistance in species with broad diets, that is, polyphagous species, as they have had an evolutionary history with a more diverse set of defensive chemistries. It also predicts that a history of feeding on some hosts—for example, groups with especially strong or diverse chemical defenses—should improve the odds of evolving insecticide resistance. We test these predictions.

## 2 METHODS

### 2.1 Data

We restricted our analysis to insect species that are pests of agriculture in the United States. This was pragmatic; we know enough about the management and ecology of the US pest fauna to do our tests. To be sure, management and reporting practices vary across countries, but this should only affect the strength of the signal of pre‐adaptation effects on insecticide resistance evolution. The core data that we needed for our tests were as follows: (i) a master list of insect and mite species that farmers in the United States have been trying to kill with synthetic insecticides, and information about their (ii) host‐plant use; (iii) phylogenetic relationships; and (iv) insecticide resistance. We were also interested in accounting for the effects of a few additional variables that could affect rates of adaptation, namely voltinism, ploidy, abundance, and pest severity. In theory, voltinism should affect the rate at which allele frequencies change, and empirically, it has been shown to have significant, albeit complex, effects on the rate of pesticide resistance evolution (Rosenheim & Tabashnik, 1990, 1991). Ploidy could also affect the rate of resistance evolution, although just how depends on the mode of resistance—for example, target‐site insensitivity versus enzymatic detoxification—and the dominance of resistance alleles (Carrière, 2003; Denholm, Cahill, Dennehy, & Horowitz, 1998). Looking at abundance should help us distinguish between the metabolic and demographic differences between host‐use generalists and specialists. And the severity of a pest, that is, its negative economic impact, should correspond to the intensity of selection pressure from insecticides.

To assemble the master list of US agricultural pests, we began with the dataset of Rosenheim et al. (1996), which covers 680 species. Then, we added to this list any species mentioned in one of the 784 regional crop profiles in the National IPM Database (http://ipmcenters.org/). This increased the total number of species examined (i.e., that we try to kill) to 902, after standardizing taxonomic names using the Global Names Resolver API (http://resolver.globalnames.org/).

Host use of these species was modeled at the level of host‐plant families, records of which came from multiple sources: for Aphidoidea (aphids), Aphids on the World's Plants (http://www.aphidsonworldsplants.info/); for Chrysomelidae (leaf beetles), Clark, 2004; for Coccoidea (scale insects), ScaleNet (http://scalenet.info, Garcìa Morales et al., 2016); for Eriophyoidea (eriophyid mites), Amrine & Stasny, 1994; for Lepidoptera (moths and butterflies), the HOSTS database (http://www.nhm.ac.uk/our-science/data/hostplants/, Robinson, Ackery, Kitching, Beccaloni, & Hernández, 2010); for Miridae (plant bugs), the Plant Bug Biodiversity Inventory (http://research.amnh.org/pbi/), via Discover Life (http://www.discoverlife.org/); for North American Hemiptera (true bugs), the Tri‐Trophic Database project (http://tcn.amnh.org/); for all pests, the CABI PlantWise fact sheets (https://www.plantwise.org/). The taxonomic scope of some of these sources is nested within the scope of others. For example, information about host use in aphids was found in three of them. In addition to these data, we included host records reported in pest profiles produced by the extension offices of land‐grant universities (e.g., http://ifas.ufl.edu/; http://www.extension.umn.edu/), although this added only a few associations. In all, these sources yielded 8,721 unique pairwise interactions between a US pest insect and a host‐plant family (Table S1). Plant‐host range size variation is given as counts of host‐plant families in Table S2. Although this table includes a few species of Orthoptera, they were excluded from the analyses, as we suspect that the host associations of these species are especially hard to characterize (all life stages are highly mobile, and it would be difficult to record an exhaustive list of what they eat).

Note that when we use taxonomic measures of diet breadth to test the pre‐adaptation hypothesis, we assume a positive correlation between taxonomic and chemical diversity. We expect this to hold, as defensive chemistry varies considerably across plant families (Kite, Grayer, Rudall, & Simmonds, 2000; Seigler, 1998). But it is a rough approximation; for example, many classes of defensive chemicals are shared across plant families (Strauss & Zangerl, 2002). One thing that could make this assumption especially dubious is intense antagonistic co‐evolution between specialist plant‐eating insects and their hosts. If that is common, the plants with the most intense and unusual defenses might exclusively host specialists, and a species that specializes on a few hosts might contend with more diverse defensive chemistries than a species with a more taxonomically diverse diet. But evidence to the contrary can be found in the nestedness of plant–herbivore trophic networks (Thébault & Fontaine, 2008). The least‐connected species in a network tend to interact with the most‐connected species; plant‐eating insects tend to specialize on commonly used host plants. Hence, reciprocal specialization between plant‐eating insects and their hosts is unlikely break the expected relationship between the taxonomic and chemical diversity of diets.

Phylogenetic relationships were estimated from published DNA sequence data. We used the PHLAWD megaphylogeny pipeline (Smith, Beaulieu, & Donoghue, 2009) to obtain a supermatrix of aligned DNA sequences, sampled from four loci (18S, COI, cytb, ef1a) and 507 of the species on the master list of US agricultural insect pests. Note that of these 507 species, we had a complete set of comparative data for only 344. From the supermatrix, we used RaxML v8.1.16 (Stamatakis, 2014) to estimate the maximum likelihood phylogeny along with the parameters of a GTR + G nucleotide substitution model (unlinked across loci). In the tree search, we imposed the NCBI taxonomy as a topological constraint. The search comprised 100 nonparametric bootstrap (BS) replications, with every fifth BS tree used as a starting tree for optimization of the un‐permuted dataset. To scale phylogenetic branch lengths to time, we used the relaxed molecular clock approach implemented in TreePL (Smith & O'Meara, 2012), which assumes an autocorrelated model of among‐lineage substitution rate variation (i.e., lineages inherit substitution rates from their ancestors) and uses penalized likelihood to find an optimal set of branch rates (Sanderson, 2002). To calibrate the divergence time estimates, we used 32 node age constraints based on previously published estimates that have been integrated by the TimeTree project (http://www.timetree.org/, Hedges, Dudley, & Kumar, 2006). The phylogeny is provided in Newick format in Appendix S1.

We drew records of insecticide resistance evolution from the Arthropod Pesticide Resistance Database (http://www.pesticideresistance.org/). We then classified each case according to the mode of action taxonomy of the Insecticide Resistance Management Committee (http://www.irac-online.org/). To be clear, we looked only at insecticides, many of which are also used to control mites, but we did not consider chemicals used only to control mites.

**Figure 1:**
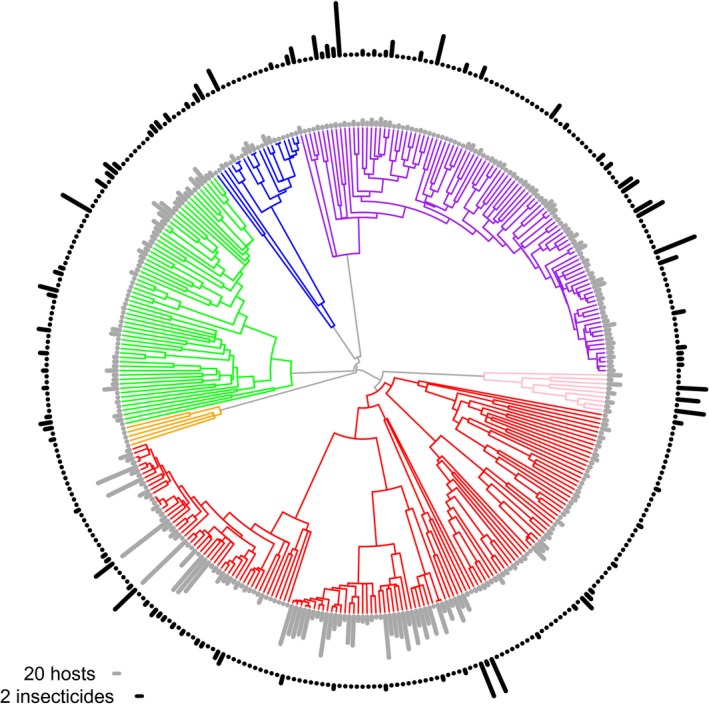
Overview of comparative phylogenetic data. Maximum likelihood estimate of time‐scaled phylogenetic relationships among insect species that are pests of agriculture in the United States, and for which we have data about their host range, insecticide resistance, ploidy, and documentation intensity. Only values for host range and insecticide resistance are shown. The difference between species in these characters is represented by the lengths of bars in concentric rings around the phylogeny: gray for host range (number of host‐plant families) and black for insecticide resistance (number of functional classes resisted). Note the scales of these bars differ by an order of magnitude. Branches are color‐coded by insect order: Coleoptera, green; Diptera, blue; Hemiptera, red; Hymenoptera, orange, Lepidoptera, purple; Thysanoptera, pink. Note that this is only a subset of the data that were analyzed by taxonomy models

Data on voltinism came mostly from the Rosenheim et al. (1996) database, with additional information for scale insects from ScaleNet, and for other species from land‐grant extension pest profiles. As in Rosenheim et al., 1996; when a range of generations per year was reported, we took the average of those values. If only a minimum number of generations per year was reported, we used the minimum. If the life cycle of a species was undocumented, but the life cycle of some of its congeners was known, we assigned to the undocumented species the average number of generations per year of its congeners. We took data on ploidy from the Tree of Sex Consortium (2014). In an attempt to distinguish between the effects of polyphagy and overall abundance (as in Ross, Hardy, Okusu, & Normark, 2013), the number of distinct geospatial records (i.e., collection events) for species of Hemiptera in the Tri‐Trophic Database was included as a covariate in analyses restricted to Hemiptera. (We lacked sufficient data to model the abundance of other groups.) Lastly, we characterized the pest severity of each species as a count of publications containing its name in the PubMed database. Note that this measure of pest severity can also be seen as a way of accounting for the documentation bias in our measures of insecticide resistance. Figure 1 provides an overview of some of the key comparative data.

### 2.2 Analysis

We took a variance decomposition approach, estimating the parameters of a variety of generalized linear mixed models. In one set of tests, we used Poisson models to explore the breadth of insecticide resistance evolution. The response variable was the count of major insecticide classes to which a pest species had evolved resistance. Predictor variables were (i) host‐plant range, measured as a count of host‐plant families for each pest species; (ii) voltinism, that is, generations per year; (iii) ploidy (diplodiploid or haplodiploid); and (iv) pest severity/documentation intensity, expressed as the number of PubMed citations referencing each species. Previous studies show us that voltinism can have curvilinear effects on insecticide resistance evolution (Rosenheim & Tabashnik, 1990, 1991). Hence, we apportioned the effects of voltinism across orthogonal linear and quadratic terms.

We used two approaches to account for the nonindependence of these variables due to shared evolutionary history. First, we used the estimated phylogenetic relationships to specify a matrix of expected covariances. Second, we specified nested random effects based on a three‐level classification (Order, Family, Genus) of pest species. We refer to the former as phylogeny models and the latter as taxonomy models. The relationships in the phylogeny models are more accurate and informative, but the taxonomy models allow us to analyze species lacking published, phylogenetically informative DNA sequence data (514 species in taxonomy models vs. 344 in phylogeny models). Moreover, the phylogeny models assume that characters evolve via Brownian motion, which could be a poor fit for characters such as host range. In contrast, the taxonomy models assume a more punctuated evolutionary model. Consistent results across phylogeny and taxonomy models would reassure us that we are not being misled by poor assumptions about the evolutionary process. In all cases, the response variable was related to the covariates through a log link function. As mentioned above, we also fit models to just the Hemiptera data that included counts of occurrence records as a covariate. We had data for 192 species for the Hemiptera taxonomy model and 129 species for the Hemiptera phylogeny model.

In a second set of tests, we explored how feeding on specific host‐plant families affects the evolution of insecticide resistance. These were phylogeny models in which the response was the number of insecticide classes to which a pest species had evolved resistance. As in the previously described models, this response varied according to a Poisson distribution. In each model, the predictors were the host range of a species, and a binary character representing use or nonuse of the focal host‐plant family. The effect of use of each of the 32 most commonly used host families was estimated in its own model. Then, we used a randomization procedure to determine the significance of the effect of each host‐plant family on the evolution of insecticide resistance. Specifically, for each host family, we estimated the effect of its use on insecticide resistance from 100 randomized datasets (host use or not‐use permuted across taxa, while keeping the empirical proportion of taxa that use each host). Then, we compared the observed effect magnitude to that null distribution.

Models were fit using Bayesian MCMC sampling with the R package MCMCglmm (Hadfield, 2010). Full prior and model specifications are given in Appendix S2. For the first set of models, MCMC analyses were run for 10–50 million iterations—long enough to ensure that all effective sample sizes were greater than 500. We used Geweke's (1991) convergence diagnostic as a check that we had sampled from the stationary distribution. Each of the host‐family models in the second set was run 10 times, with 10 million iterations for each run. Convergence for these models was assessed with both the Geweke diagnostic and that of Gelman and Rubin (1992).

## 3 RESULTS

### 3.1 Do pests with more diverse diets evolve resistance to more diverse insecticides?

We had a full complement of phylogenetic and comparative data for 344 species (Figure 1). These represent six insect orders: 145 species of Hemiptera, 101 species of Lepidoptera, 63 species of Coleoptera, 21 species of Diptera, 9 species of Thysanoptera, and 5 species of Hymenoptera. Note that because of the constraints imposed on the estimate, only the relationships among taxa within a taxonomic rank were free to vary, for example, the relationships among species in a genus.

After accounting for phylogenetic history and differences in voltinism, ploidy, and pest severity, we found that diet breadth predicts the evolution of insecticide resistance (Table 1). In both the phylogeny and taxonomy models, host range had a significant positive effect on the number of resisted insecticide classes. The same was true of pest severity. As per previous studies, we found concave effects from voltinism on the evolution of resistance, that is, the most positive effects when voltinism was neither too small nor too great. The effects of ploidy were not statistically significant.

**Table 1:** Summaries of models examining the effects of host range on insecticide resistance evolution

	Effect	Sample size	pMCMC
All pests phylogeny model			
Diet breadth (# host‐plant families)	0.56	48435	0.00012***
Voltinism (# generations per year)	10.14	48781	0.029*
Voltinism^2^	−8.01	50000	0.0011**
Ploidy (diplodiploidy vs. haplodiploidy)	−0.056	50000	0.95
Documentation intensity (# PubMed publications)	0.15	48938	2e‐05***
All pests taxonomy model			
Diet breadth (# host‐plant families)	0.49	79732	8.89e‐05***
Voltinism (# generations per year)	6.34	81042	0.2
Voltinism^2^	−12.14	87980	0.00027***
Ploidy (diplodiploidy vs. haplodiploidy)	−0.47	80906	0.39
Documentation intensity (# PubMed publications)	0.41	85861	<1e‐05***
Hemiptera phylogeny model			
Diet breadth (# host‐plant families)	0.52	40487	0.046*
Voltinism (# generations per year)	−0.15	43494	0.97
Voltinism^2^	−1.46	49990	0.56
Ploidy (diplodiploidy vs. haplodiploidy)	0.82	48521	0.56
Abundance (# collection events)	0.055	40986	0.82
Documentation intensity (# PubMed publications)	0.27	51421	0.089
Hemiptera taxonomy model			
Diet breadth (# host‐plant families)	0.59	7856	0.034*
Voltinism (# generations per year)	1.11	8664	0.81
Voltinism^2^	−3.33	9000	0.3
Ploidy (diplodiploidy vs. haplodiploidy)	−0.62	9000	0.41
Abundance (# collection events)	−0.1	9000	0.72
Documentation intensity (# PubMed publications)	0.32	8492	0.055

The effect size given is the mean of the posterior distribution and is on a log scale, the sample size is the effective sampling of that parameter by the MCMC analysis, and pMCMC is a Bayesian analog of the frequentist *p*‐value. In these models, the response variable is the number of insecticide classes to which a pest insect species has evolved resistance. In addition to the main analyses, we looked at Hemiptera only models, for which we could include the number of distinct collection events for each species as a proxy for abundance. Statistical significance thresholds are denoted with symbols: * is < 0.05, ** is < than 0.01, *** is < than 0.0001

The species with the highest documentation intensity was the tobacco hornworm, *Manduca sexta*. In part, this reflects that it is an important pest. But this is also due to the fact that it is a model system in insect physiology and neurobiology. To make sure that this did not bias our inferences, we repeated our analyses with *M. sexta* excluded. The sign, magnitude, and significance of estimated effects were unaltered (results not shown).

In both the phylogeny and taxonomy models restricted to Hemiptera but including the abundance of each species as a covariate, we found the same effect from host range: positive on the breadth of insecticide resistance (Table 1). Abundance, measured as a count of collection events, was not significant, nor were the effects of voltinism.

### 3.2 Does feeding on particular host‐plant groups improve the odds of evolving insecticide resistance?

**Figure 2:**
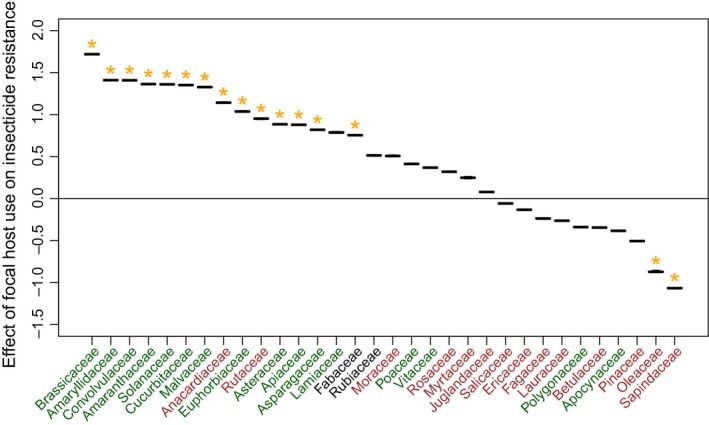
Effects of feeding on the 32 most common host families on the evolution of insecticide resistance. The *Y*‐axis shows the estimated effect of use of the focal host‐plant family on a Poisson parameter predicting the number of insecticide classes resisted. Statistically significant effects are denoted with orange stars and were determined by comparing the empirical values with those calculated from 100 randomized datasets. Plant family names are colored according to predominant growth form: green for herbaceous, brown for woody, and black for a more even mix of both major growth forms

Insect use of 14 of the 32 most common host‐plant families was associated with significantly increased insecticide resistance (Figure 2). Only two host‐plant families (Oleaceae and Sapindaceae) were associated with significantly lower levels of insecticide resistance.

## 4 DISCUSSION

The pre‐adaptation hypothesis is corroborated by correlations between host‐use and insecticide resistance evolution. It would appear that feeding on some plant groups boosts the odds of evolving resistance to insecticides. And plant‐eating insect species with more diverse diets are apt to evolve resistance to more diverse insecticides.

What kinds of hosts increase the odds of insecticide resistance evolution? We did not a priori characterize the differences between host‐plant families; we expected that some families would be different from others, without knowing the particulars. But Figure 2 shows a marked split between herbs and trees. With a few exceptions, feeding on plant families with typically herbaceous growth forms increases insecticide resistance, whereas feeding on plant families with typically woody growth forms does not. According to the plant apparency hypothesis, a plant's defensive strategy should be a function of how easy it is for herbivores to find (Agrawal, 2007; Silvertown & Dodd, 1996). High apparency species (such as ecologically dominant tree species) will use quantitatively acting traits, such as high concentrations of nutrition‐inhibiting tannins. These are essentially brute force defenses that can be thwarted with more brute force. For example, leaf tannins can reduce the efficiency with which insects convert leaf matter to body mass, but this can be overcome by eating more leaf tissue, and allocating more energy to digestion (Barbehenn et al., 2009). Harder‐to‐find species (such as ephemeral herbs) will use qualitative defenses, such as low doses of exotic toxins. It is exposure to these exotic toxins that are thought to pre‐adapt a plant‐eating insect to evolve insecticide resistance. Hence, the split we find between the effects of herbs and trees supports both the pre‐adaptation hypothesis and the plant apparency hypothesis. Of course, this does not rule out alternative explanations; the relationships we find between plant‐feeding and insecticide resistance are mostly consistent with the pre‐adaptation and plant apparency hypotheses, but leave the door open for alternative hypotheses.

One such alternative hypothesis would be that insects that eat herbs are more likely to evolve insecticide resistance because herbs are treated with more insecticides. To rule this out, we looked at insecticide applications across the 22 top‐value crops in California in 2012. Using data from the Pesticide Action Network (PAN) Pesticide Database (http://www.pesticideinfo.org/) and the USDA National Agricultural Statistics Service, we calculated the number of pesticide applications per year for a given acre of each crop (Table S5). We found no significant difference between how many times per year insecticides are applied to woody (*n *=* *9, mean = 2.87 ± 1.37) and herbaceous crops (*n *=* *13, mean = 3.21 ± 3.1). (The main driver of insecticide use was crop value, which accounted for 54% of the variation in a simple linear regression.) The influence of growth form on insecticide resistance evolution does not seem to stem from variation in selection pressure.

Now let us turn to what this study tells us about the effect of diet breadth on insecticide resistance evolution. It is striking that the pre‐adaptation hypothesis makes good predictions even when we ignore the population genetic factors that we typically use to predict the evolution of insecticide resistance (e.g., Georghiou & Taylor, 1977). Examples of such factors are the size of populations, the dominance and fitness costs of resistance alleles, the heterogeneity of environments, and the rate at which individuals migrate into and out of treated fields. To be fair, population genetic models built from these factors might capture some of the key differences between host‐use generalists and specialists. For example, increasing the diet breadth of a species is apt to increase its effective population size (Sisterson et al., 2004). And increasing diet breadth could also effectively increase the flow of genetic variation into treated fields, as it increases the chances that areas near a treated field will contain suitable host species, and support source populations for migration. (As an aside, the effects of this increased gene flow are complex, and its influence on resistance evolution is hard to predict (Caprio & Tabashnik, 1992; Carrière et al., 2012).) Thus, population genetic models surely have provided us with some insight into how diet breadth affects resistance evolution. But population genetic models have not captured other potentially important differences between generalists and specialists.

The pre‐adaptation hypothesis assumes important differences in metabolic capacity: More polyphagous individuals will have more ways of detoxifying xenobiotics, and more chances that one will be a lucky solution to a new problem. It is also possible that, in more polyphagous species, the genetic architectures for detoxification lend themselves to greater evolvability (Hansen, 2003; Hardy, 2017; Janz & Nylin, 2008). For example, gene expression networks may be more modular, or gene families may be more diverse and open to functional divergence. Diet breadth could also change the evolvability of resistance by affecting neutral demographic processes, for example, that rate at which a newly treated area is first colonized (Forister & Jenkins, 2017; Normark & Johnson, 2011). Appreciation of these differences could be critical for the sound management of insecticide resistance.

We can find more explicit theoretical studies of how niche breadth governs adaptation outside of the insecticide resistance literature. For example, Whitlock (1996) modeled the rate at which a population adapts to a particular environment as a function of the probability at which that environment is encountered. In our case, the chance that an individual in a population will be in a treated field should be higher in a population of crop specialists than in a population of host‐use generalists. This exposure probability affects the rate at which resistance alleles are fixed in a population (Whitlock, 1996). Furthermore, larger populations—as we might expect for generalists—fix beneficial mutations more slowly than smaller ones (Kimura & Ohta, 1969). This might lead us to conclude that resistance evolution should be slower in generalists. But note that these models pertain to the rate at which alleles are fixed (or purged) from a population. This rate may be of little use in managing the evolution of insecticide resistance, which can take place without the fixation of alleles.

This study also shows us something about the modes of insecticide resistance. The pre‐adaptation hypothesis assumes metabolic resistance; plant defensive molecules are degraded by insect enzymes. But that is only one possibility. Others are target‐site insensitivity, behavioral changes, and physical exclusion. In principle, resistance to multiple types of insecticides could be due to target‐site mutations at multiple loci, or the evolution of one general‐purpose mechanism for excluding xenobiotics. Multilocus target‐site insensitivity may be just as likely to evolve in a specialist as a generalist. But the other modes of multiresistance seem more apt to evolve in generalists. The support we find for the pre‐adaptation hypothesis suggests that these generalist‐specific modes of resistance are important enough to produce a detectable signal in the midst of the noise created by a myriad of other factors shaping resistance evolution (including target‐site insensitivity‐based resistance).

Before we conclude, let us stress that our findings are contingent on the many assumptions we have made about evolutionary processes. They depend on our assumption of a correspondence between the taxonomic and chemical diversity of set of host plants. They also depend on the recorded observations of insecticide resistance being a decent representation of reality. We suspect that many cases of insecticide resistance are simply not reported. We attempted to account for documentation intensity in our models, but maybe these efforts were inadequate. To try and understand the evolution of insecticide resistance, we have used what we know. But what we know is imperfect. Let us also stress that our findings could be explained in other ways. The antithesis of the pre‐adaptation hypothesis would be that differences in recent selection intensity are sufficient to explain the observed differences in resistance evolution across species—ancient history has nothing to do with it. Generalists evolve more resistance not because they are more pre‐adapted, but because they tend to be more severe pests, which are exposed to more insecticides. We could call this the Markovian‐selection hypothesis. But as our analyses accounted for pest severity, this explanation seems unlikely. What makes it even more unlikely is our finding that resistance evolution is greater in species that feed on herbaceous than woody hosts, as we apply insecticides as liberally to herbaceous and woody crops. There may be plausible alternatives to the pre‐adaptation hypothesis, but the Markovian‐selection hypothesis is probably not one of them.

## 5 CONCLUSIONS

Under the right selective environment, it seems that insects will evolve resistance to any insecticide. On the other hand, most of the insect species considered as agricultural pests in the United States have not evolved any kind of resistance—at least that has been documented in the literature. And many others have evolved resistance to only some of the insecticides they encounter. This suggests that our problems with insecticide resistance could be much worse and that there is much to lose by not better understanding the process of insecticide resistance evolution. One upshot of this study is that generalists may be especially prone to evolving insecticide resistance. Another is that this is not something we could have learned from standard population genetic models. Not all pests are the same; their specific evolutionary histories and biologies can have a big impact on their potential to evolve insecticide resistance.

## DATA ARCHIVING

Data for this study are available as Supplementary Documents.
